# Bee Venom Effect on Glioblastoma Cells Viability and Gelatinase Secretion

**DOI:** 10.3389/fnins.2022.792970

**Published:** 2022-02-11

**Authors:** Agata Małek, Joanna Kocot, Kamila Mitrowska, Andrzej Posyniak, Jacek Kurzepa

**Affiliations:** ^1^Department of Medical Chemistry, Medical University of Lublin, Lublin, Poland; ^2^Department of Pharmacology and Toxicology, National Veterinary Research Institute, Puławy, Poland

**Keywords:** bee venom, glioblastoma, anticancer potential, MMP-2, MMP-9

## Abstract

**Background:**

The involvement of MMP-2 and MMP-9 in the pathogenesis of various kinds of cancers including glioblastoma is well documented. The evaluation of the anticancer potential of honey bee (*Apis mellifera*) venom (BV) consisting of the inhibition of MMP-2 and MMP-9 secretion in a glioblastoma cell culture model was the aim of the study.

**Methods:**

8-MG-BA and GAMG human primary glioblastoma cell lines vs. HT-22 mouse hippocampal neuronal cells were applied for the study. The BV dose (0.5, 1.0, 1.25, 1.5, 1.75, 2.0, 2.5, and 5.0 μg/ml) and time-dependent (24, 48, 72 h) cytotoxicity was evaluated with the tetrazolium-based colorimetric assay (MTT test). MMP-2 and MMP-9 activities in the cell culture medium under different BV concentrations were determined by gelatin zymography.

**Results:**

A dose and time-dependent BV effect on cytotoxicity of both glioblastoma cell lines and hippocampus line was observed. The weakest, but statistically important effect was exerted by BV on HT-22 cells. The greatest cytotoxic effect of BV was observed on the 8-MG-BA line, where a statistically significant reduction in viability was observed at the lowest BV dose and the shortest incubation time. The reduction of both gelatinases secretion was observed at 8-MG-BA and GAMG lines without significant effect of HT-22 cell line.

**Conclusion:**

*In vitro* studies indicate that BV has both cytotoxic and inhibitory effects on the secretion of MMP-2 and MMP-9 in selected lines of glioma, suggesting anticancer properties of BV.

## Introduction

Clinically, the classification of gliomas includes four grades, with grade four glioblastoma representing the most malignant type and also known as glioblastoma multiforme (GBM) (Atiq and Parhar, 2020). GBM is a highly aggressive brain tumor. Different grade GBMs are the most frequent primary malignant brain tumors (15% of all intracranial neoplasms and up to 50% of all primary malignant brain tumors) (Linhares et al., 2020). Despite significant progress in GBM treatment research, it remains a great therapeutic challenge. Current treatment methods include surgical tumor resection with chemotherapy and radiotherapy thereafter. However, even with advanced surgical techniques such as e.g., fluorescence-guided resection, the complete removal of cancer cells is almost impossible because tumor cells located at the edges of tumors in perivascular niches remain in most cases (Atiq and Parhar, 2020). Therefore, the new kind of therapy together with the applying of various natural compounds seems to be an interesting research topic that gives hope for finding an effective treatment for glioblastoma. Bee venom (BV) is a biotoxin (apitoxin) synthesized and secreted by a venom gland placed in the abdominal cavity of a bee. BV is a complex mixture containing several biologically active components with a variety of pharmaceutical properties (Rady et al., 2017; Carpena et al., 2020). The most prevalent component of BV is a representative polypeptide group—melittin, composed of 26 amino acids. Phospholipase A_2_ (PLA_2_) is believed to be the second most abundant one (Choo et al., 2010; Sciani et al., 2010; Lee et al., 2016), while the third component is apamine (Rady et al., 2017). In BV samples collected from honey bees (*Apis mellifera*) in Poland, the content of the mentioned substances has been amounted to 61.15–70.15% (average 64.40%) for melittin, 11.24–15.05% (average 13.00%) for PLA_2_ and 2.09–4.18% (average 3.10%) for apamine (Rybak-Chmielewska and Szczȩsna, 2006).

The anticancer properties or the ability to prevent the chemotherapy-induced side effects of BV as well as its selective components have already been reported. Jamasbi et al. (2018) have revealed the effectiveness of melittin against gastric cancer cells. The authors compared the monomeric form of this peptide with its dimer form and found the former being more cytotoxic at low concentrations (1–5 μM), while at higher concentrations (10 μM) the cytotoxic effect of both forms was comparable. Kim et al. (2015), in turn, proved that PLA_2_ could prevent inflammatory responses in cisplatin-induced acute kidney injury. According to Zhou et al. (2013) both melittin and apamine have been shown to trigger apoptotic cell death in hepatocellular carcinoma cells (HepG2). Zheng et al. (2015) in turn, suggested that BV could inhibit colon cancer cell growth, and its antiproliferative effect may be related to the induction of apoptosis by activation of DR4 and DR5 and inhibition of NF-κB signaling pathway.

When considering the therapeutic properties of BV components against the tumor located within the Central Nervous System (CNS), their ability to penetrate the blood-brain barrier (BBB) should be especially considered. Earlier studies show that both apamine and melittin have the ability to cross the BBB, which allows the BV components to be considered as chemical compounds with potential therapeutic properties exhibiting activity within the CNS (Upadhyay, 2014; Wehbe et al., 2019).

Matrix metalloproteinases (MMPs), including gelatinases (MMP-2 and MMP-9), exert pleiotropic effects under pathological and physiological conditions (Boguszewska-Czubara et al., 2019; Drankowska et al., 2019). MMPs also play a key role in cancer invasion and metastasis. Many studies have reported elevated MMPs expression and/or activity in cancer cells that metastasize to distant organs including the lungs, liver, lymph nodes, or adrenal medullary (Yelken et al., 2017). MMP-9 has been considerably involved in glioblastoma progression. Its overexpression correlates with increased invasive glioma grades, whereas a decrease of MMP-9 expression is associated with favorable outcome and response to Temozolomide treatment (Quesnel et al., 2020). Zhang et al. (2019) reported the positive expression of MMP-2 in glioma was closely related to the tumor diameter, severity of peritumoral edema, degree of enhancement, and pathological grade of tumor observed in magnetic resonance imaging (MRI). In addition, MMP-2 was highly expressed in brain glioma, and it was considered as a negative factor for prognosis (Zhang et al., 2019).

In the available literature data, studies evaluating the ability of BV to inhibit glioblastoma cell growth and metastasis are scarce. Considering this fact, the aim of the current study was to examine the *in vitro* influence of BV on cell viability and the BV induced secretion of MMP-2 and MMP-9 by glioblastoma cell lines (GAMG, 8-MG-BA) vs. hippocampal cells (HT-22).

## Materials and Methods

### Bee Venom

Samples of *Apis mellifera* bee venom were collected from a private apiary located in Siedliszcze (Lublin region, Eastern Poland), by stimulating the bees with electric current pulses using Bee Venom Collector BVC 5.0 2017 (IGK Electronics Ltd., Varna, Bulgaria). The general scheme of bee venom collection procedure was illustrated by in Ref. (Carpena et al., 2020). The samples collected between May and September 2017 were pooled together and stored in the dark at 5°C until analysis (Rybak-Chmielewska and Szczȩsna, 2006; Kokot et al., 2009). The bee venom stock solution of 1 mg/ml in PBS was prepared, vortexed for 1 min, sonicated for 10 min, and filtered (Matysiak et al., 2011). The stock solutions were prepared directly before each experiment and then diluted in a complete medium to obtain the required concentration.

### The Determination of Melittin Content in the Bee Venom Sample

The determination of melittin content was performed in Department of Pharmacology and Toxicology of the National Veterinary Research Institute (Poland) by liquid chromatography with tandem mass spectrometry (LC-MS/MS) according to the method of Zhou et al. (2010) with some modifications. Briefly, the bee venom sample (2 mg) was weighed and dissolved in 1 ml of water followed by vortexing for 5 min. Next, the tube was centrifuged at 10,000 rpm at 4°C for 10 min. The supernatant was filtered through a 0.22-μm nylon syringe filter and diluted 100 times with 0.1% formic acid before the LC-MS/MS analysis. For separation and identification of melittin in BV extract, an ExionLC AC system consisting of a binary gradient pump, autosampler, column oven, and system controller coupled to QTRAP 5,500 linear ion trap quadrupole mass spectrometer (AB Sciex) with electrospray ionization (ESI) interface was used. Data acquisition and analysis were accomplished with Analyst 1.6.3. Mass spectrometric analysis was performed in positive ESI mode. The following instrument conditions were used: ion source temperature 300°C, curtain gas 20 psi, nebulizer gas 50 psi, heater gas 60 psi, ion spray voltage 3,500 V. Quantitation of analyte was performed by monitoring the following SRM transitions: m/z 712.8–> 70.0, collision energy 140 V with dwell time 100 ms. The chromatographic separation was performed on a Kinetex octadecyl analytical column (2.6 μm, 150 × 2.1 mm) with an octadecyl guard cartridge (Phenomenex, Torrance, CA, United States). The mobile phase A consisted of 0.1% formic acid in acetonitrile, while the mobile phase B contains 0.1% formic acid in water. Linear gradient steps were used with initial conditions set at 5% of the mobile phase A, held for 0.5 min, increasing to 95% from 0.51 to 2.00 min, and then returned to the initial composition of the mobile phase A (5%) from 2.01 to 5.00 min. The flow rate of the mobile phase was 0.25 ml/min. The column temperature was 30°C and the injection volume was 5 μl.

### Cell Culture

The human glioblastoma cell lines were obtained from Leibniz Institute, DSMZ—German Collection of Microorganisms and Cells Cultures GmbH (GAMG—ACC 242; 8-MG-BA—ACC 432). As control cells, immortalized mouse hippocampal cell line, HT-22 (Sigma Aldrich, Saint Louis, MO, United States) was used. The cells were cultured in Dulbecco’s Modified Eagle Medium (DMEM), GAMG and HT-22 and in Eagle’s Minimum Essential Medium (EMEM)—8-MG-BA, supplemented with 10% (v/v) fetal bovine serum, penicillin (10,000 U/ml), streptomycin (10 mg/ml) and amphotericin B (250 μg/ml). The cells were incubated at 37°C, 5% CO_2_ atmosphere. The cells were maintained in the logarithmic growth phase by regular passage at 80% confluence.

### Cell Viability Assay

The cytotoxicity of the bee venom was studied against human glioblastoma cell lines (GAMG and 8-MG-BA) vs. neuronal (hippocampal) cell line (HT-22) used as a control line. After 24-h incubation in growth medium with an addition of 10% fetal bovine serum (FBS) on 96-well plates, the cells were treated with the following concentrations of bee venom: 0 (vehicle), 0.5, 1.0, 1.25, 1.5, 1.75, 2.0, 2.5, and 5.0 μg/ml in medium without FBS. The cells were cultured at 37°C in the presence of 5% CO_2_-air for the next 24, 48, and 72 h. The bee venom cytotoxicity was evaluated using the MTT colorimetric method based on the ability of viable cells to the transformation of yellow, soluble tetrazolium salts [3-(4,5-dimethylthiazol-2-yl)-2,5-diphenyltetrazolium bromide, MTT] to purple insoluble formazan, by cellular dehydrogenases. After incubation with bee venom solution, cell cultures were supplemented with 10 μl per well of 5 mg/ml MTT (Sigma-Aldrich, Saint Louis, MO, United States) stock in PBS, and the incubation was continued for 4 h at 37°C. Next, the medium with MTT was removed, and the formed crystals were dissolved in 100 μl of DMSO. The solution absorbance was measured at 570 nm, using a spectrophotometric plate reader Epoch, BioTek Instruments (Vermont, United States). The relative cytotoxic activity was determined as the amount of bee venom capable of reducing 50% of cell viability (IC50 value). The experiment was performed three times with triplicates for each concentration.

### Analysis of Matrix Metalloproteinase-2 and Matrix Metalloproteinase-9 Activity in Cell Culture Supernatants

After 72-h incubation with the following concentration of BV 1.0, 1.5, 2.0, 2.5, 5.0 μl/ml, the media from the cells were collected to measure the secreted MMP-2 and MMP-9 activities. The concentrations of BV used for MMPs analysis as well as the 72 h incubation time were chosen on the base of the cell viability assay. The experiment was performed three times with duplicates for each concentration (*n* = 6). MMP-2 and MMP-9 activities were evaluated with the use of gelatin zymography according to previously applied methods (Golab et al., 2014). Briefly, 80 μl of cell culture media was mixed with 20 μL of sample loading buffer containing 10% sodium dodecyl sulfate (SDS) and incubated for 30 min at room temp. Next, the proteins were separated by polyacrylamide gel electrophoresis (PAGE) on a 10% gel supplemented with 0.05% gelatin type A from porcine skin; G2500 (Sigma-Aldrich, St. Louis, MO, United States). After electrophoresis, the gels were washed with 2.5% Triton X-100 three times for 20 min each to remove SDS. Next, 48-h-incubation was performed at 37°C in the buffer pH 7.2 containing 1% Triton X-100. The gels were stained with the solution containing 0.1% Coomassie Blue R-250, 20% methanol, and 10% glacial acetic acid in distilled water and destained in 10% solution of acetic acid thereafter. The MMP-2 and MMP-9 were detected as colorless bands (digested gelatin) on a blue background (undigested gelatin). Zymography allows detecting both pro-active (latent) and active forms of MMPs as the SDS is used to activate non-proteolytic pro-MMPs into MMPs with catalytical activity without changing their molecular mass. The enzymes were identified by comparing their localization with molecular mass standards (SM0441) (Fermentas Life Sciences, St. Leon-Rot, Germany). Zymographic gels were scanned and quantified with ImageJ software (National Institute of Health, Bethesda, MD, United States). The activities of MMP-2 and MMP-9 were expressed as the optical density (OD) of the substrate lysis zone.

### Statistical Analysis

Statistical analysis was performed using GraphPad Prism 8 software. Non-linear regression (curve fit) was used to establish IC50 values of BV after 24, 48, and 72 h of incubation for all three cell lines. The results of cells viability were expressed as mean values as well as standard deviation, and the statistical significance of the differences between the control vehicle and the other groups of each three cell lines in 3 time points was evaluated using a two-way analysis of variance (ANOVA) followed by Tukey’s *post-hoc* test. Values were considered significant with *p* < 0.05.

## Results

### Melittin Content in Bee Venom

**FIGURE 1:**
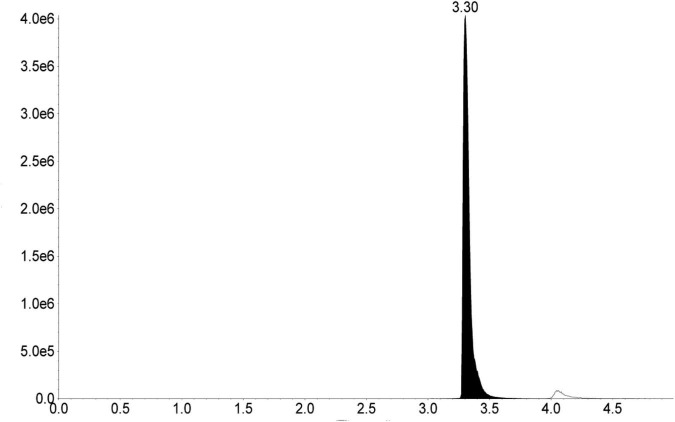
MRM chromatogram of melittin (m/z 712.8–> 70.0) in the bee venom sample.

Melittin content in the bee venom sample was found to be 69.0 ± 0.1% of dry weight (Figure 1).

### The Effect of Bee Venom on Cells Viability *in vitro* (IC50 Estimation)

**FIGURE 2:**
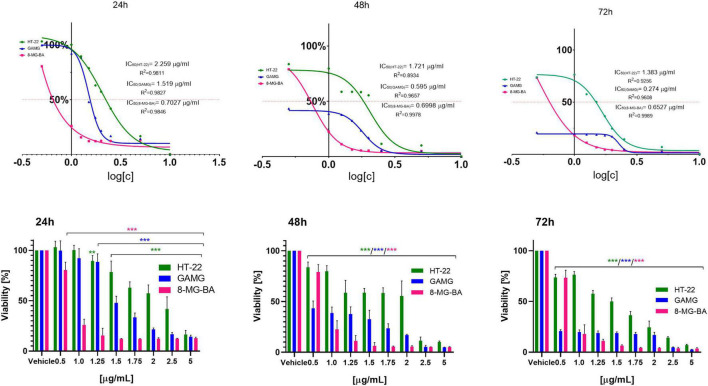
The IC50 values (μg/ml) and cells viability (%) regarding GAMG, 8-MG-BA and HT-22 cells after 24, 48, and 72 h of incubation with BV; **p < 0.01, ***p < 0.001 vs. GAMG, 8-MG-BA and HT-22 vehicle, respectively; Tukey test.

To analyze the effect of BV on cells viability, the inhibitory concentration of BV (IC50) was assessed depending on the incubation time; 24, 48, and 72 h. The most significant time-dependent effect of BV was observed on GAMG cells, where the increase of incubation time from 24 to 72 h decreased the IC50 from 1.519 to 0.274 ng/ml (more than 5-fold reduction in BV dose when incubated for 72 vs. 24 h). The lowest value of BV IC50 after 24 h incubation was noticed for 8-MG-BA cells (0.7027 ng/ml). IC50 for these cells did not change significantly after longer incubation times (0.6998 and 0.6527 ng/ml after 48 and 72 h, respectively). The highest IC50 value of BV was noticed for HT-22 cells at each of the time intervals, suggesting the low sensitivity of these cells to BV. The increase of incubation time from 24 to 72 h resulted in the decrease of IC50 value for HT-22 from 2.259 ng/ml to 1.383 ng/ml for HT-22 cells. All results were expressed in Figure 2.

#### The Bee Venom Effect on Matrix Metalloproteinase-2 and Matrix Metalloproteinase-9 Secretion

**FIGURE 3:**
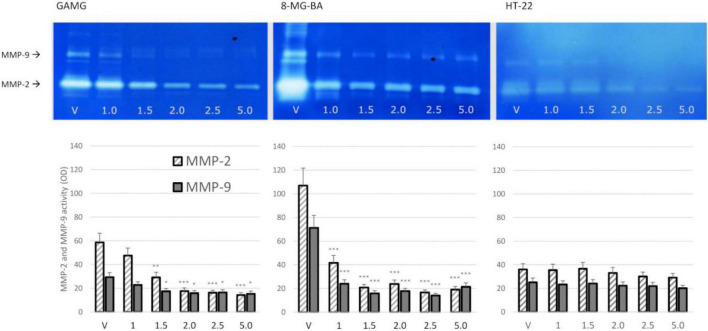
BV effect on MMP-2 and MMP-9 secretion from GAMG, 8-MG-BA, and HT-22 cells. Significant decrease of gelatinase secretion was observed in GAMG and 8-MG-BA cells after 72 h of incubation with different concentrations of BV. The effect of BV on MMP-2 and MMP-9 secretion from HT-22 cells did not reach statistical significance. ANOVA with Tukey *post-hoc* test. *p < 0.05, **p < 0.01, ***p < 0.01 compared to controls (V, vehiculum).

The dose-dependent effect of BV on MMP-2 and MMP-9 secretion from GAMG and 8-MG-BA cells was revealed. After 72 h of incubation with BV at concentration 1.5 μl/ml and higher, the significant reduction of MMP-2 and MMP-9 secretion from GAMG cells was noticed. Similar inhibitory effect was observed on 8-MG-BA cells, however, a statistical significant reduction of gelatinase secretion was already observed at BV concentration of 1.0 μl/ml. There was no significantly visible effect of BV on MMP-2 and MMP-9 secretion from HT-22 cells at any analyzed concentrations. The representative zymograms were shown in Figure 3.

## Discussion

Among the various agents that influence the development, progression, and metastasis of cancer, there are many natural compounds that have pleiotropic properties and possess chemopreventive potential, e.g., soybean isoflavones, curcumin, retinoids, resveratrol, epigallocatechin or cannabinoids. Some effects of them are well documented in the literature, for example, curcumin, resveratrol are known as strong antioxidants. The anticancer potential of the remaining compounds requires further research, as is the case with, for example, cannabinoids; animal studies showed that the activation of cannabinoid receptors 1 and 2 by tetrahydrocannabinol (THC) results in impaired proliferation and invasion of cancer cells, induces apoptosis (accumulation of ceramides in culture), and further reduces the tumor volume (McAllister et al., 2005; Galanti et al., 2008; Marcu et al., 2010; Abrams, 2016). Therapeutic administration of bee venom to treat various diseases, initially primarily arthritis or ailments related to joints and muscles, has been used in traditional Chinese medicine from 1,000 to 3,000 BC (Zhang et al., 2018). The application of BV is widespread in the treatment of not only immune-mediated diseases but also cancer. Both *in vitro* and *in vivo* studies have confirmed that the main BV constituent, melittin, is responsible not only for the cytotoxic effect, but also for the immunomodulatory and proapoptotic effect against various types of cancer cells (Oršolić, 2012). BV has been shown to have an antiproliferative effect on cancer cells via several mechanisms. It induces apoptosis through the activation of death DR4 and DR5 death receptors. In addition, melittin reduces tumor growth and metastasis (Liu et al., 2002). BV inhibits cancer cells growing also due to the activation of caspase 3 and 9 pathways and inhibition of NF-κB pathway signaling, leading to inhibition of the expression of proliferative and antiapoptotic genes encoding Bcl-2, cyclooxygenase-2 (COX-2), cellular inhibitor of apoptosis 2 (cIAP-2), inducible nitric oxide synthase (iNOS) and cytosolic phospholipase A2 (cPLA2) (Park et al., 2014).

Finally, it is worth emphasizing that the potential of BV in the treatment of SARS-CoV-2 infections has also recently been recognized. It has been noted that in Wuhan, China, beekeepers, as a group exposed to frequent bee bites, are more resistant to COVID-19 infection, probably due to regulation of the immune response, which can cause an increase in the titer of IgE and IgG antibodies (Kasozi et al., 2020).

The obtained results showed a statistically significant effect of unfractionated bee venom on the viability of glioblastoma cells and physiological hippocampal cells. The observed effect was different for each cell type tested. Earlier reports indicated different chemosensitivity of glioblastoma cell lines (Wolff et al., 1999). Glioblastoma cell malignancy and drug sensitivity are related to the cell of origin; nervous stem cell-like origin causes higher malignancy and drug sensitivity than others (Jiang et al., 2017). After an incubation time of 24 h, 8-MG-BA was proved to be more sensitive cells to BV effects than other tested cell lines; during each applied concentration of BV, the viability of 8-MG-BA cells was the lowest among the analyzed configurations. Moreover, the IC50 for BV was the lowest after 24 h incubation with respect to 8-MG-BA cells. However, after 48 h of incubation, the viability of GAMG cells significantly decreased. The IC50 for GAMG decreased threefold compared to the 24 h incubation and was less than the IC50 for 8-MG-BA after 48 h incubation. Extending the incubation time to 72 h did not significantly affect the IC50 value for 8-MG-BA cells, however, a decrease in IC50 for GAMG cells was observed at this time of incubation. When GAMG cells were incubated with bee venom for longer periods of time, we observe a significant decrease in cell viability even at lower concentrations compared to shorter incubation times. Thus, the longer the incubation the significantly greater the cytotoxic effect against GAMG cells without the need for higher doses of BV. For GAMG cell line, the incubation time of bee venom also proved to be a significant factor affecting cell viability, and by modifying the treatment time we can obtain a satisfactory effect even at lower doses of active substance. Summarizing this part, the studies showed the dose-dependent and time-dependent sensitivity of the tested cells to bee venom.

The glial cells were more sensitive to BV effects than the nerve cells of the hippocampus. For higher concentrations and/or longer incubation times with bee venom of HT-22 line cells, a reduction in the viability of hippocampal cells used as a control was also observed. Thus, bee venom may exhibit some inhibitory effects on neuronal cell viability in the brain. It is therefore worth emphasizing the necessity of caution in the selection of appropriate doses that will simultaneously be effective against tumor cells and will not significantly affect the viability of neuronal cells, so as not to impair their essential functions that they perform in numerous processes.

Substrate-zymography, applied in this study, still represents the most simple, sensitive, and quantifiable assay for MMPs analysis, which is able to identify, simultaneously in the same sample, the entire panel of enzymes that are capable of degrading a specific substrate. The identification of gelatinase activity is possible by using gelatin as a substrate (gelatin zymography). Moreover, zymography showing the activity of MMPs, but not directly the amount of protein, provides additional information on the activity of these enzymes in the tested sample (Lowrey et al., 2008; Ricci et al., 2016).

The study also showed a dose-dependent effect of bee venom on the secretion and activities of MMP-2 and MMP-9 in the culture medium. When analyzing the activity of both gelatinases in GAMG cells, depending on the dose of the BV used for incubation, a gradual reduction in activity can be observed. A statistically significant reduction in MMP-2 and MMP-9 activity was obtained at concentrations higher than 1 mg/ml. The reduction in the activity of gelatinases may be explained simply by the reduced expression and secretion of these enzymes to the extracellular space under the influence of BV. The observed decrease in the activity of MMP-2 and MMP-9 cannot be explained by the decreased cell viability, as cell death under the influence of BV would lead to the release of intracellular reservoirs of MMP-2 and MMP-9 into the medium. In such cases, it could even result in an increase in the activity of these enzymes.

The active ingredients of BV have the ability to pass through the BBB and can therefore be used in the treatment of diseases of the CNS (Gu et al., 2020). BV has proven to be an effective treatment in animal models of Alzheimer’s Disease, Parkinson’s Disease, Epilepsy, Multiple Sclerosis, and Amyotrophic Lateral Sclerosis (Silva et al., 2015). The sensitivity of several cancer cells, including renal, lung, liver, prostate, bladder, and mammary cancer cells to BV peptides such as melittin and phospholipase A2 together with the known permeability of these peptides through BBB allows to hope that BV may also be active against cancer located in the CNS (Oršolić, 2012).

## Conclusion

In conclusion, the performed studies showed a dose and time-dependent effect of unfractionated bee venom on the survival of neoplastic cells of glial origin. Moreover, an inhibitory effect on the secretion of both gelatinases was demonstrated, which may have a potential impact on tumor spread.

Further research should focus on elucidating the molecular mechanism of the observed effect and on identifying the active components of the venom exhibiting these properties.

## Data Availability Statement

The raw data supporting the conclusions of this article will be made available by the authors, without undue reservation.

## Author Contributions

JKu, AM, and JKo participated in research design. AM, KM, and AP conducted to the experiments. AM, JKu, KM, and AP wrote and contributed to the writing of the manuscript. JKu supervised the work. JKu and JKo initiated the research. All authors contributed to the article and approved the submitted version.

## Conflict of Interest

The authors declare that the research was conducted in the absence of any commercial or financial relationships that could be construed as a potential conflict of interest.

## Publisher’s Note

All claims expressed in this article are solely those of the authors and do not necessarily represent those of their affiliated organizations, or those of the publisher, the editors and the reviewers. Any product that may be evaluated in this article, or claim that may be made by its manufacturer, is not guaranteed or endorsed by the publisher.
